# Synteny analysis of genes and distribution of loci controlling oil content and fatty acid profile based on QTL alignment map in *Brassica napus*

**DOI:** 10.1186/s12864-017-4176-6

**Published:** 2017-10-12

**Authors:** Nadia Raboanatahiry, Hongbo Chao, Liangxing Guo, Jianping Gan, Jun Xiang, Mingli Yan, Libin Zhang, Longjiang Yu, Maoteng Li

**Affiliations:** 1 Department of Biotechnology, College of Life Science and Technology, Key Laboratory of Molecular Biology, Huazhong University of Science and Technology, Wuhan, 430074 China; 2 Hubei Collaborative Innovation Center for the Characteristic Resources Exploitation of Dabie Mountains, Huanggang Normal University, Huanggang, 435599 China; 3 School of Life Science, Hunan University of Science and Technology, Xiangtan, 411201 China

**Keywords:** *Brassica napus*, QTL alignment map, Seed oil alteration, QTL, Candidate genes, Synteny, Gene network interaction, Metabolism pathway

## Abstract

**Background:**

Deciphering the genetic architecture of a species is a good way to understand its evolutionary history, but also to tailor its profile for breeding elite cultivars with desirable traits. Aligning QTLs from diverse population in one map and utilizing it for comparison, but also as a basis for multiple analyses assure a stronger evidence to understand the genetic system related to a given phenotype.

**Results:**

In this study, 439 genes involved in fatty acid (FA) and triacylglycerol (TAG) biosyntheses were identified in *Brassica napus. B. napus* genome showed mixed gene loss and insertion compared to *B. rapa* and *B. oleracea,* and C genome had more inserted genes. Identified QTLs for oil (OC-QTLs) and fatty acids (FA-QTLs) from nine reported populations were projected on the physical map of the reference genome *“Darmor-bzh”* to generate a map. Thus, 335 FA-QTLs and OC-QTLs could be highlighted and 82 QTLs were overlapping. Chromosome C3 contained 22 overlapping QTLs with all trait studied except for C18:3. In total, 218 candidate genes which were potentially involved in FA and TAG were identified in 162 QTLs confidence intervals and some of them might affect many traits. Also, 76 among these candidate genes were found inside 57 overlapping QTLs, and candidate genes for oil content were in majority (61/76 genes). Then, sixteen genes were found in overlapping QTLs involving three populations, and the remaining 60 genes were found in overlapping QTLs of two populations. Interaction network and pathway analysis of these candidate genes indicated ten genes that might have strong influence over the other genes that control fatty acids and oil formation.

**Conclusion:**

The present results provided new information for genetic basis of FA and TAG formation in *B. napus.* A map including QTLs from numerous populations was built, which could serve as reference to study the genome profile of *B. napus,* and new potential genes emerged which might affect seed oil. New useful tracks were showed for the selection of population or/and selection of interesting genes for breeding improvement purpose.

**Electronic supplementary material:**

The online version of this article (10.1186/s12864-017-4176-6) contains supplementary material, which is available to authorized users.

## Background

Dissection of the genetic architecture of a species is one of the best approach to understand its identity, evolution history and allow the understanding of the genetic network mechanism that run the entire organization [1–3]. Each gene has an important role within this organization, which might affect one phenotypic trait, or in case of pleiotropy, affect several unrelated traits [4, 5]. Hunting specific genes for agriculturally and economically valuable traits is needed. Nowadays, breeding of cultivar with high oil content and advantageous fatty acid profile has become a necessity, since the demand in oil has increased with the growing population [6, 7].

The rapeseed (*Brassica napus*) is a well-known source of vegetable oil and is the preferred oil crop for biodiesel production in Europe [8], the most important breeding goal is to increase the oil content, since 1 % increase of seed oil content is equivalent to 2.3–2.5% increase in seed yield in *B. napus* [9]. The allotetraploid *B. napus* (A^n^A^n^C^n^C^n^, 2n = 38) was derived from hybridization of *B. rapa* (A^r^A^r^, 2n = 20) and *B. oleracea* (C^o^C^o^, 2n = 18). Long years of evolution and artificial selection have made the A^n^ and C^n^ genomes of *B. napus* somewhat different from the A^r^ genome of *B. rapa* and the C^o^ genome of *B. oleracea* [10]. The *Brassica* genera is closely related to the model plant *Arabidopsis thaliana*, the divergence occurred approximately 14 to 20 million years ago [11, 12]. In the evolution history of *Brassicaceae* family, *Brassica* species underwent a whole genome triplication event compared to *A. thaliana*, which promoted their speciation, and that event was followed by genome duplication and rearrangement events [13–16]. *A. thaliana* genome could be subdivided into 24 blocks and 21 among them have been conserved in *B. napus* [17, 18]. Great opportunities are opened to undertake multiple important studies for further exploitation or improvement of this crop with the release of *B. napus* genome sequence in 2014 [19]. In our knowledge, the distribution of all genes involved in oil formation in *B. napus* was not reported before.

Seed oil is mostly composed of triacylglycerol (TAG), which represents 35% of seed weight in *A. thaliana* [20, 21]. The pathway of TAG biosynthesis leading to the oil formation has been elucidated in plant, and some key genes have been identified [21–25]. Li-Beisson et al. (2013) revealed that at least, 120 enzymatic reactions and more than 600 genes encoding the proteins and regulatory factors, were involved in acyl-lipid formation in *A. thaliana* [21]. Besides, combination of multiple genes, influenced by the environment has been demonstrated to control seed oil content trait [26, 27]. Also, many studies have shown that oil content and fatty acid profile influenced each other [28–30]. Understanding the genes architecture and network that control the variation of seed oil composition allows a better insight to get the desired profile according to the final usage. For instance, high unsaturated fatty acid (UFA) oil has been recommended for food preparation, because it allows rapid cooking time and less oil absorption [31]. Otherwise, many genes have been cloned and showed their effectiveness on oil content improvement. For example, expression of rapeseed DGAT increased oil content in *A. thaliana* [32], and BnGPDH and BnGPAT increased oil content of ~4% in transgenic seeds [33].

Quantitative trait loci (QTL) analysis is a powerful tool for genetic investigation in order to identify loci responsible for the variation of phenotypic trait. The phenotypic traits are usually valuable traits in agriculture and economy. QTL is extensively used in plant breeding. Because QTLs correlate with variation of phenotype, the corresponding loci could be amplified and consequently are expected to improve the phenotype. Multiple oil related QTLs have been discovered in different populations of *B. napus* [28, 34–49], but the number and location of QTLs in different populations varied a lot, which needs their unification in one map for comparison. Establishing a unique map combining QTLs from multiple populations would be of a great utility, which could facilitate the comparison of these QTLs. Importantly, if QTLs of multiple populations with same environment overlaps in one region, they could be defined as fixed QTLs, otherwise, they would be population specified QTLs. However, building a consensus map is challenging since the position of QTLs varies with population, and the markers used for the studies are different, so their comparison or their unification into one consensus map is rather difficult [50]. Liu et al. (2016) built a map aligning QTLs for oil content QTLs (OC-QTLs) from six populations of *B. napus* by projection on the physical map of the reference genome *“Darmor-bzh”*. One-hundred and ten QTLs could be positioned and 53 among them were overlapping [51]. Besides, the determination of QTLs coupled with the identification of associated candidate genes would permit the comprehension of these genes authority over traits [52, 53].

The purposes of the present study were as follows: (1) identify the genes involved in FA and TAG biosyntheses in *B. rapa, B. oleracea* and *B. napus,* and analyze the genes synteny; (2) compare the FA-QTLs and OC-QTLs from different genetic mapping populations of *B. napus* by construction of a map aligning QTLs; (3) identify the relative candidate genes in the confidential intervals of QTLs and analyze their interaction and metabolism pathway. Our study revealed a map of FA-QTLs and OC-QTLs, with diverse populations, showing fixed and specified QTLs, and additionally highlighted new potential genes that might affect seed oil content and FA composition.

## Results

### Gene synteny analysis revealed higher gene copy number in *B. napus*

**Fig. 1:**
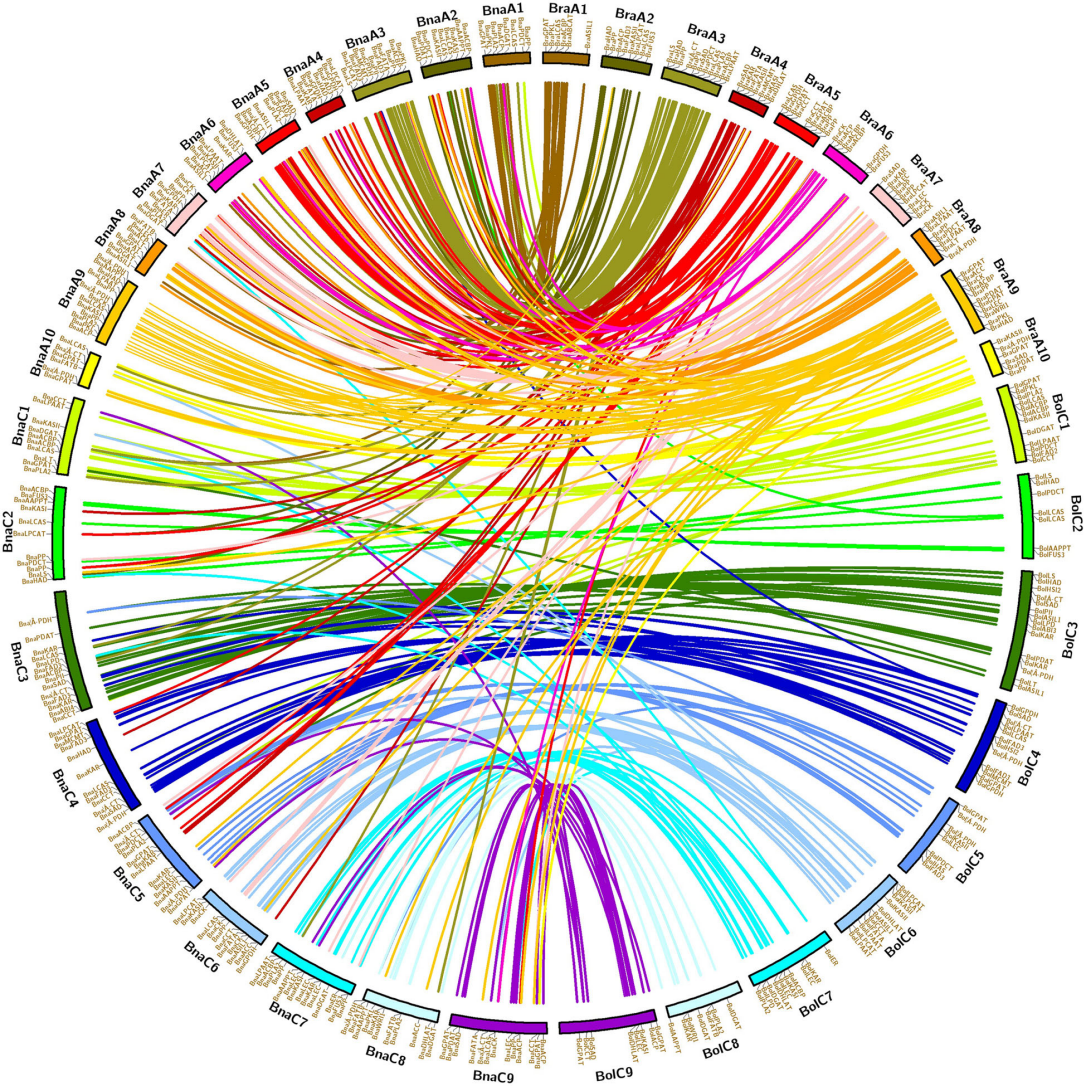
Synteny analysis of genes in *B. rapa, B. oleracea* and *B. napus*. The map was built with Circos software. Bra, Bol and Bna represent chromosomes in *B. rapa, B. oleracea and B. napus*, respectively

In total, 439 genes related to FA and TAG biosyntheses were identified in the genome of *B. napus*, they were homologous to 110, 224 and 173 genes from *A. thaliana, B. rapa* and *B. oleracea*, respectively. In *B. napus*, A^n^ and C^n^ genomes contained 220 and 219 genes, respectively (Table 1; Additional file 1: Table S1). The genes synteny in *B. rapa, B. oleracea* and *B. napus* are illustrated on Fig. 1, it was found that genes were mostly located on A3 and C3 chromosomes. Obviously, the number of inherited genes greatly increased in *B. napus*, and some of them were not maintained in the same chromosome location as their parents. While observing the synteny between them, it was revealed that the genes could be lost or inserted on the genome. In fact, 27 genes were lost in total, 17 of them were lost in A^n^ chromosomes and 10 were lost in C^n^ chromosomes. This is the case of KASII genes Bra014202 and Bra014203 on A^r^8, and Bol012577 on C^o^6 and Bol042053 on C^o^7, which did not have descendant genes in *B. napus*. They thus had sequence similarity with the other KASII genes found in *B. napus*. However, seven lost genes were replaced in the other genome, i.e. genes lost in A^n^ were replaced in C^n^, and reversely; so, five genes on A^n^ genome were replaced on C^n^ genome, and two genes on C^n^ genome were replaced on A^n^ genome. For instance, *B. rapa* DHLAT Bra006486 on A^r^3 should have been transmitted on A^n^3, but was replaced on C^n^8 in *B. napus* (BnaC08g03220D). Besides, 69 genes were inserted, of which 14 were found on A^n^ chromosomes, and 55 were found on C^n^ chromosomes. For example, PLA2 Bra039011 (A^r^7) in *B. rapa* had homologous BnaA07g01090D (A^n^7) and BnaC07g01540D on C^n^7 in *B. napus*, which accounted for one gene insertion on C^n^ genome.

**Table 1:** Distribution of genes involved in FA and oil biosynthesis in *Brassica*

Chromosomes	*B. rapa*	*B. napus*	Chromosomes	*B. oleracea*	*B. napus*
A01	21	21	C01	22	19
A02	13	15	C02	10	14
A03	38	34	C03	31	35
A04	17	11	C04	23	23
A05	31	28	C05	19	24
A06	15	17	C06	16	18
A07	21	23	C07	22	23
A08	20	16	C08	12	13
A09	35	27	C09	15	21
A10	10	12			
Scaffold	3	0	Scaffold	3	0
An	0	16	Cn	0	29
Total	224	220	Total	173	219

Counting the number of lost and inserted genes, 42 additional genes were found in *B. napus* compared to *B. rapa* and *B. oleracea*. They originated from gene duplication or triplication which mostly appear in other chromosomes. For example, β-PDH gene Bra032361 on A^r^9 in *B. rapa*, had two descendant genes in *B. napus*, which were BnaA08g17570D on A^n^8 and BnaA09g26420D on A^n^9. Also, three copies of PP genes were found on C^n^ chromosomes, BnaC01g22010D on C^n^1, BnaC07g31970D on C^n^7 and BnaCnng22260D on an unknown C^n^ chromosome, and they were homologues of *B. rapa* PP gene Bra006653 on A^r^3. Note that *B. napus* PDAT gene BnaC03g53840D on C^n^3 had no parental homologue, this gene was homologue of *A. thaliana* PDAT gene At3g44830. These results showed higher amount of gene copy in *Brassica* compared to *A. thaliana*, and also confirmed the fact that *B. napus* genome showed mixed of gene loss and insertion compared to *B. rapa* and *B. oleracea* genomes. Moreover, distribution on chromosomes of all genes involved in oil formation in *B. napus* were highlighted in our findings.

### Identified overlapping QTLs on the reference genome *“Darmor-bzh”*

**Fig. 2:**
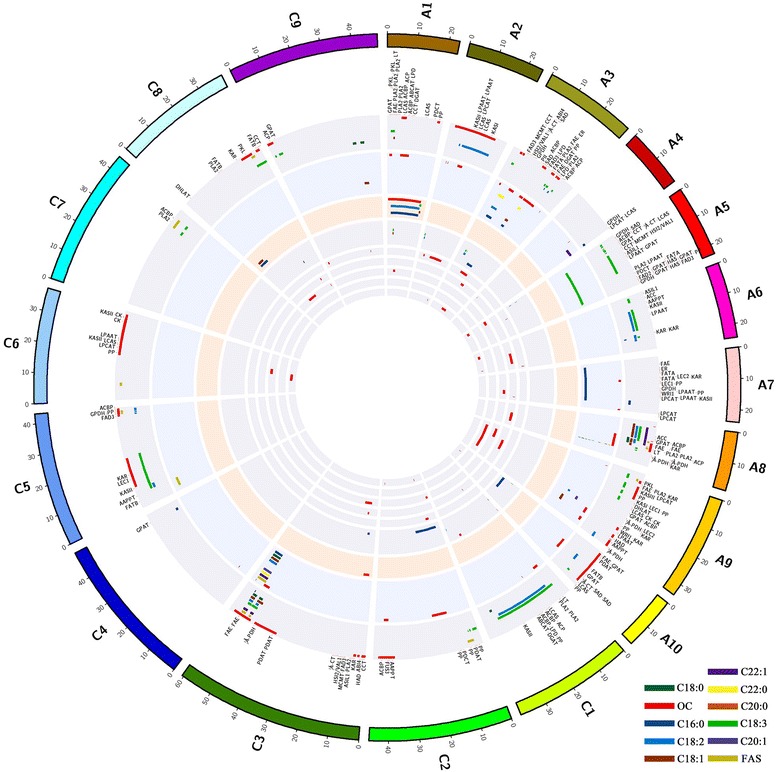
Map representing QTL alignment for FA and OC traits in nine populations of *B. napus* with their related candidate genes. From inside to outside, the nine inner circles with background color represent 9 populations (*DY, M201xM202, SG, RNSL, Z5, PT, SO, TN* and *KN* respectively), and short bars with color within the 9 inner circles represent QTLs identified in different populations and linkage groups. The blocks at the outermost circle represent the 19 genetic linkage groups. The gene labels between the outermost circle and second circle show the candidate genes and their position

**Fig. 3:**
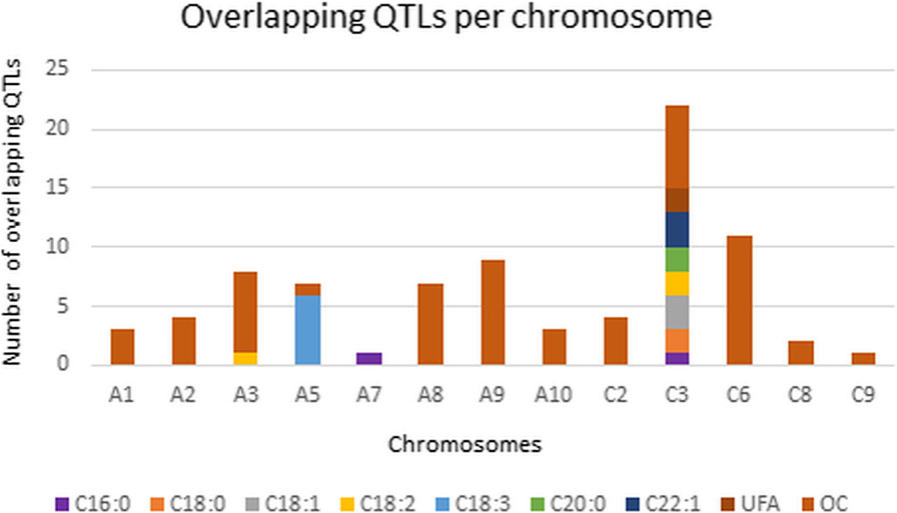
Proportion of identified overlapping QTLs

By using of E-PCR, 217 molecular markers could be settled on the physical map of *“Darmor-bzh”* emphasizing 335 FA-QTLs and OC-QTLs from *DY, KN, M201 × M202, PT, RNSL, SG, SO, TN* and *Z5* populations. The detailed information about these QTLs with their respective physical location are presented on Additional file 2: Table S2. The QTL alignment map related to these QTLs is illustrated on Fig. 2 and the proportion of overlapping QTLs per chromosome are represented on Fig. 3. It was observed that 82 overlapping QTLs were found widespread in all chromosomes unless A4, A6, C1, C4 and C7 (Fig. 3, Additional file 3: Table S3). Obviously, 64 overlapping QTLs were from two populations (e.g: *qOC-A2–1-KN1* and *qOC-A2–2-TN* were overlapping on A2), 15 overlapping QTLs were from three populations (e.g: *qOC-A8–1-TN, qOC-A8-RNSL* and *qOC-A8–3-KN* were overlapping on A8), 3 overlapping QTLs from four populations were also observed (e.g: *qOC-A3-Z5, qOC-A3-DY, qOC-A3–3-TN* and *qOC-A3–5-KN* were overlapping on A3).

OC-QTLs overlapped the most in this study (59/82 overlapping QTLs), followed by C18:3 (five overlapping QTLs), and then C18:1, C18:2 and C22:1 (three overlapping QTLs each), and finally the saturated fatty acids and UFA (two overlapping QTLs each). Chromosome C3 hosted the highest number of overlapping QTLs (22) with all trait studied except for C18:3.

While observing the QTL positions between populations, QTLs from *TN* and *KN* populations often overlapped. *TN* and *KN* populations had in common Wuhan and Shanxi, China as environments. Considering the overlapping QTLs according to their environments (*PT* was cultivated in Canada; *DY, RNSL* and *SO* were cultivated in Europe, *KN, TN, M201 × M202* and *Z5* were cultivated in China, and *SG* was cultivated in Europe and China), it was unclear to delimit fixed QTL region according to these environments because overlapping QTLs were of populations with dissimilar environments, especially those with three and four populations. For example, in the region where *qOC-A1-SO, qOC-A1-SG, qOC-A1–2-KN* and *qOC-A1–3-TN* overlapped on A1, these populations were both developed in Europe and China. However, regions could be observed in overlapping QTLs involving two populations. Thus, 43 among these 82 overlapping QTLs might be fixed QTLs for Chinese environments. No fixed QTLs for Europe were found, also because SG population was cultivated both in China and Europe. The remaining 39 overlapping QTLs were then of mixed population. The Canadian cultivated population *PT’* QTLs overlapping once with that of *TN* population, and appeared as the only co-localization with other populations’ QTLs. The genetic architecture of this *PT* population might be very different from the others. These results confirmed that genotype and environment influenced QTLs, which in turn affected the detected overlapping QTLs of our map. Also, fixed QTL regions for particular environment could be identified with our approach (e.g. Chinese environment).

### Potential candidate genes identified in QTLs regions

**Fig. 4:**
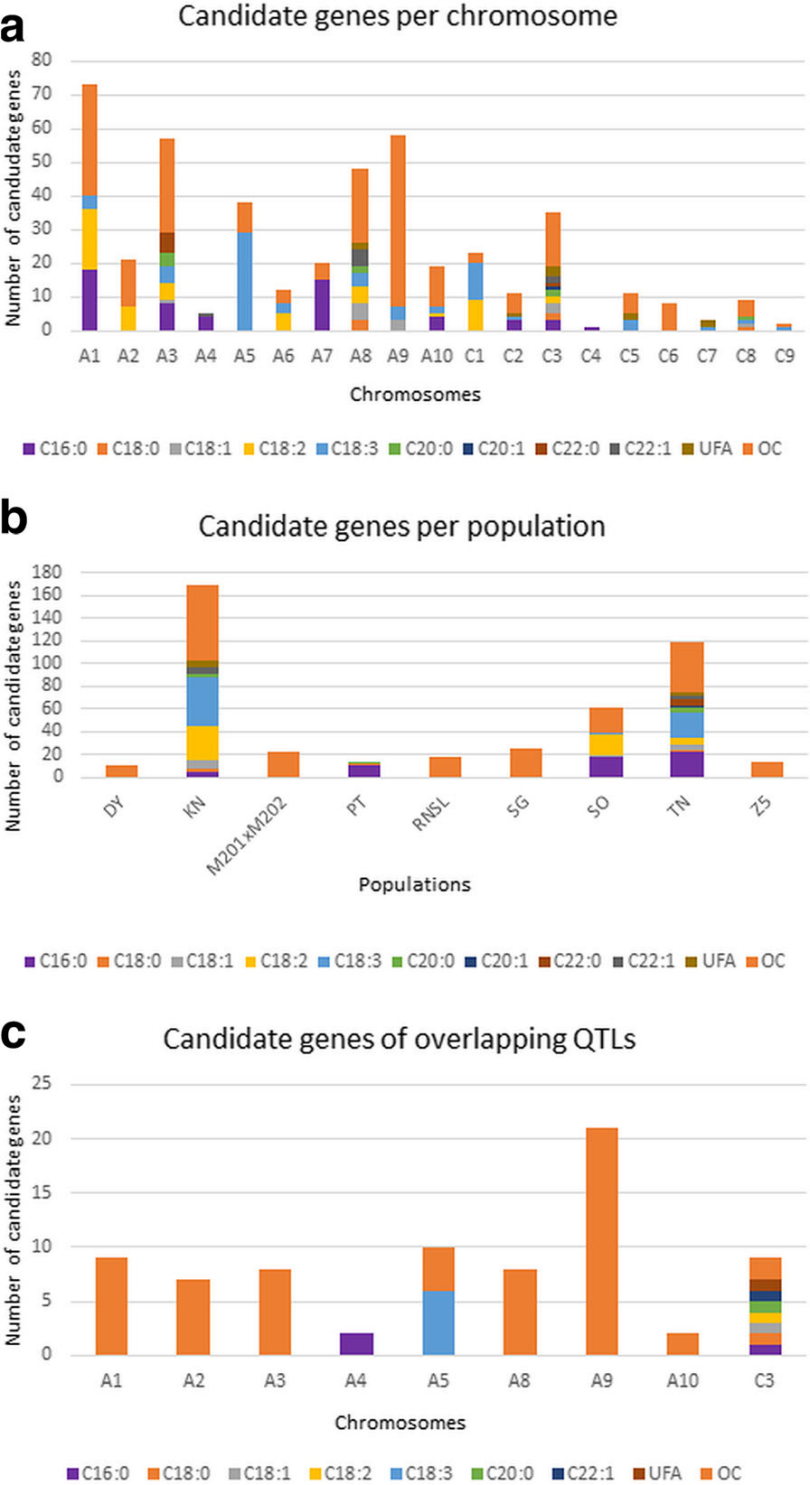
Proportion of identified candidate genes. **a** Candidate genes identified in each chromosome. **b** Candidate genes identified in each population. **c** Candidate genes identified in overlapping QTLs

A total of 218 among the 439 genes which were mentioned above, were identified as candidate genes in 162 QTLs intervals (Additional file 4: Table S4). The proportion of candidate genes in each chromosome, in each population and in overlapping QTLs are illustrated on Fig. 4. Obviously, the highest amount of candidate genes detected was for oil content trait. Besides, it was discovered that some candidate genes could be found in many QTL intervals and also, they might affect more than one trait, for example, KASI gene BnaA02g24400D was located in QTL interval of C18:2-QTL (*qC18:2-A2–3-KN*) and OC-QTLs (*qOC-A2–4-KN* and *qOC-A2–1-Z5*). Considering the number of candidate genes for each trait in each chromosome, OC-QTLs had 148 candidate genes detected and 70 remaining candidate genes were for FA traits. These candidate genes were mainly detected in QTLs intervals of *TN* and *KN* populations. Otherwise, 76 among these 218 detected candidate genes were observed in 57 overlapping QTLs. They were in majority candidate genes for oil content (61/76 genes). Sixteen genes were found in overlapping QTLs involving three populations, and the remaining 60 genes were found in overlapping QTLs of two populations (Fig. 4).

The detected candidate genes varied with populations, some of these detected candidate genes might affect more than one trait and one trait might be affected by multiple genes. For instance, FAE gene BnaC03g65980D on C3 might affect all studied traits unless C18:3. Further analysis revealed that these 218 candidate genes belonged to 45 families. One trait could be affected by many gene families, as in OC traits, but the most frequent gene families for each trait were PLA2 (C16:0, C18:2, UFA and OC), FAE (C18:0, C18:1, C18:2, C20:0, C20:1, C22:0, C22:1 and UFA), ACBP and LCAS (C18:2), GPAT (C18:3), PP (C20:1), CT, FAD3, HIS2/VAL1, LPD and MCMT (C22:0), respectively. Obviously, FAE which are involved in FA elongation, emerging long chain fatty acid at the expense of C18:X, might affect all studied traits unless for C18:3. Our findings indicated candidate genes that possibly have influence over multiple traits.

### Candidate genes interaction network and metabolic pathway analyses

**Fig. 5:**
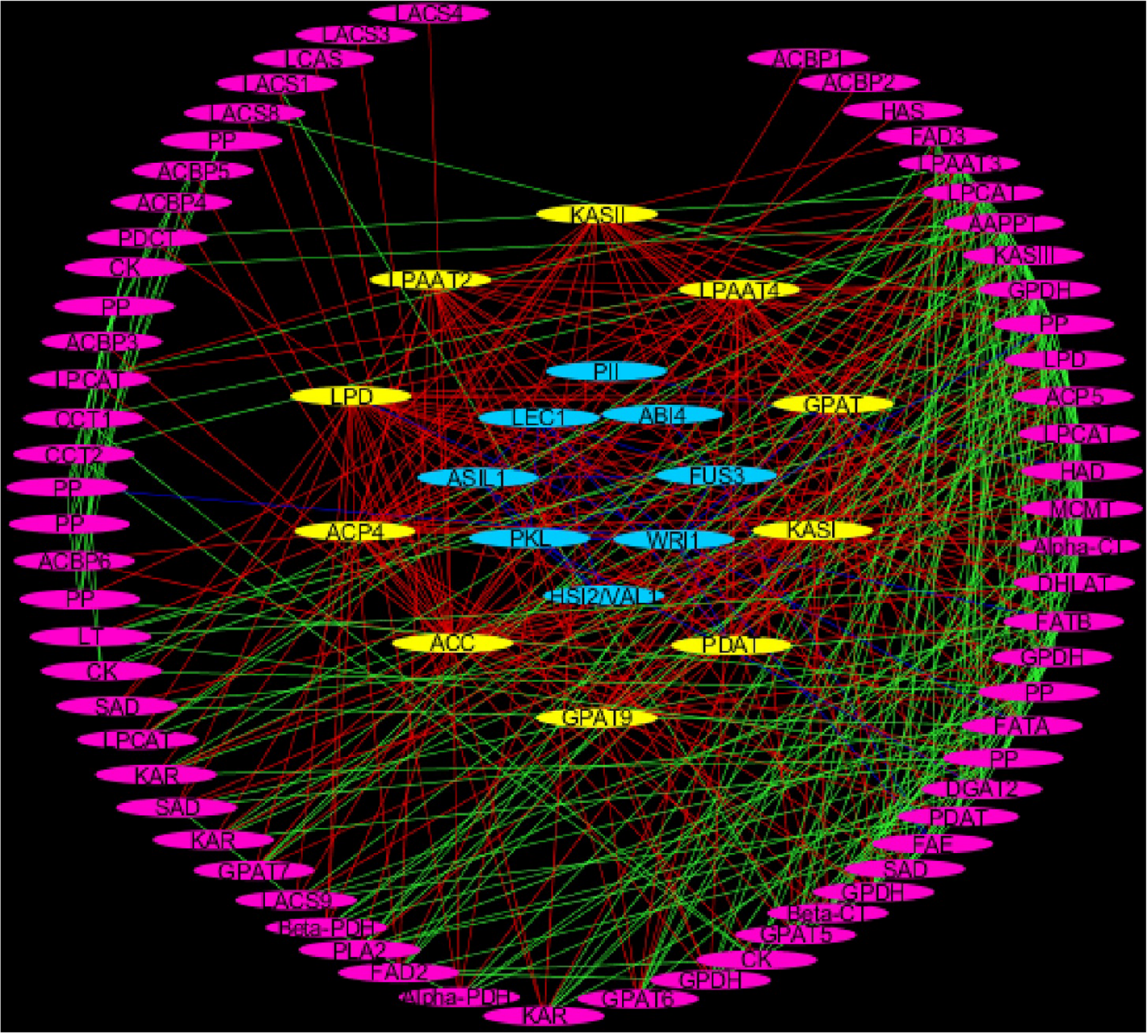
Candidate genes interaction network associated with fatty acid synthesis and oil formation. The analysis was made with orthologous *A. thaliana* genes by using STRING (http://string-db.org/) and visualized with Cytoscape_V3.2.1. 83 nodes and 413 edges are shown. Purple nodes indicate genes involved in FA and TAG biosyntheses. Yellow nodes with their related red edges represent the genes that interact the most with the other genes (DL≧20). Blue nodes with their related blue edges represent transcription factors

In order to understand the interaction between candidate genes, we analyzed interaction network and pathway which involved them in oil formation and FA synthesis. The interaction analysis was made with STRING, and visualized with Cytoscape_V3.2.1. Because *B. napus* and *B. oleracea* are still not available on STRING Database, we used orthologous genes in *A. thaliana* to perform the analysis (Additional file 4: Table S4). Thus, 91 genes from *A. thaliana* were used for this study. The results indicated 83 nodes and 413 edges (Fig. 5). It was surprising that the transcription factors (ABI4, ASIL1, FUS3, HSI2/VAL1, LEC1, PKL, PII, WRI1) interacted poorly with the rest of the genes (few edges connected them with the other genes). However, ten genes belonging to six families, which were ACC, ACP, GPAT, KAS, LPAAT and LPD, interacted the most with the other genes (DL ≥ 20). They might have more influence over the other genes. These ten genes were found within 57 QTLs intervals and might affect C16:0, C18:1, C18:2, C18:3, C22:1 and OC (Additional file 4: Table S4). LPD, ACC, ACP and KAS are plastidial key enzymes which have important roles in FA biosynthesis. LPD is an enzyme that contributes to the transformation of pyruvate into acetyl-CoA by decarboxylation. ACC is an enzyme that catalyzes the carboxylation of acetyl-CoA to produce malonyl-CoA. ACP conveys the growing FA chain between enzymatic domains of fatty acid synthase (FAS) during biosynthesis. KAS are enzymes involved in FA elongation. GPAT and LPAAT are key enzymes that work in endoplasmic reticulum. GPAT catalyzes the conversion of G3P to LPA. LPAAT is an enzyme that catalyze the acylation of LPA into PA. FAE gene family which was previously found in QTL interval of all traits unless C18:3, seemed not have high interaction with the other genes. LPD, ACC, ACP and KAS were influential candidate genes for the above-mentioned traits which indicated that these traits were affected at earlier stage of FA biosynthesis. However, GPAT and LPAAT genes were also highly connected to the other genes, which indicated that traits could be affected at multiple level of the oil biosynthesis.

**Fig. 6:**
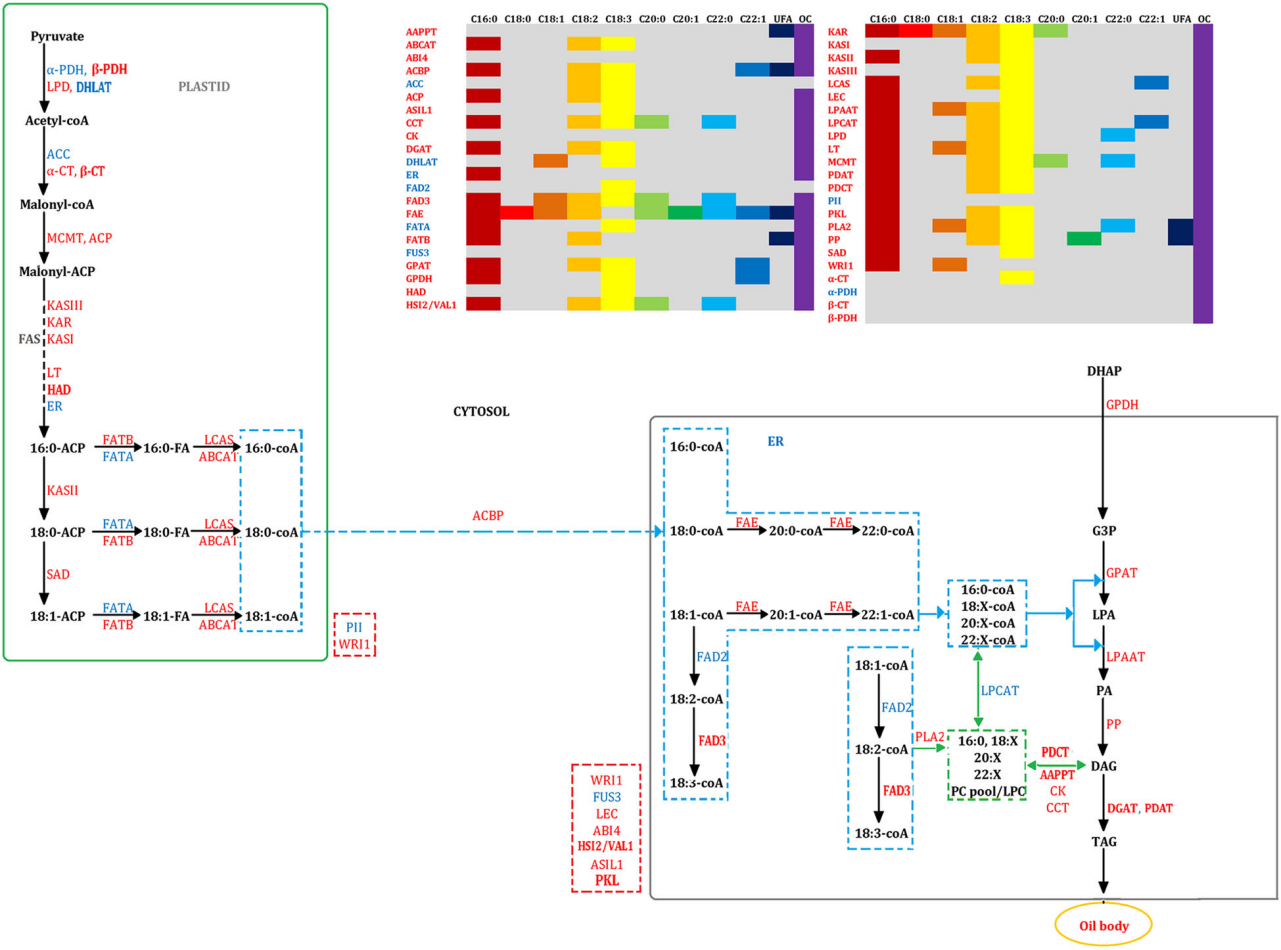
Metabolic pathway of candidate genes related to fatty acid biosynthesis and oil formation. Metabolic pathway was constructed referring to the Acyl-Lipid Metabolism of The *Arabidopsis* Book and the website ARALIP (http://aralip.plantbiology.msu.edu/). On the left, genes in blue and in red represent the candidate genes detected in this study. Genes in blue are the genes detected in QTLs of single populations and genes in red are those detected in QTLs of multiple populations. Transcription factors are inside the red dashed rectangular area. At the top right, the candidate genes were arranged with the traits that might be affected. Each trait are represented with different color, grey area means that the trait could not be affected by the gene

The metabolic pathway for fatty acid and TAG biosyntheses is shown on Figs. 6, 37 gene families were observed in QTLs from multiple populations, for example, ACBP genes were seen in OC-QTLs of all nine populations. The remaining eight gene families were observed in QTLs in single population, for example, ACC genes were observed only in C18:2, C18:3 and C22:1 QTLs of *KN* population. All genes could affect oil content unless ACC and FAD2 genes. Most of genes could affect C16:0, C18:2 and C18:3, with 29, 24 and 32 gene families involved, respectively (Fig. 6 and Additional file 4: Table S4). Earlier, we found that genes of KASI, KASII, LPAAT, GPAT, PDAT, ACC, ACP interacted most with other genes, which might be dependent of them. These genes were found in QTLs of multiple populations unless PDAT and ACC. Moreover, they could affect oil content traits unless ACC genes. Our findings indicated that traits could be affected at multilevel of oil biosynthesis.

## Discussion

### The evolutionary history of *Brassicaceae* was reflected in the present study

While identifying the genes involved in FA biosynthesis and TAG formation, 439 genes were found in *B. napus* and they were homologous to 110 genes of *A. thaliana,* 224 genes of *B. rapa* and 173 genes of *B. oleracea*. Multiple copies of genes belonging to unique family were perceived in both *A. thaliana* and *Brassica* species. The genes might belong to the same class, presenting a slight dissimilarity in amino acid sequence, or to different classes showing significant difference in structure (e.g. protein domain), but conserve the core structure that label them as a part of the family, like the ACBP genes [54].

It was discovered that *Brassica* species have experienced a whole genome triplication (WGT) event that occurred possibly while diverging from *Arabidopsis* [14, 16]. Thus, *Brassica* genome should have three times the size of *Arabidopsis* genome, and the number of genes and chromosomes as well should be three times higher than those of *Arabidopsis*. WGT was followed by chromosome restructuration, translocation, fusion, or recombination of precursor chromosomes that led to the reduction of chromosome number in *Brassica* [13, 15, 16]. Consequently, pressure of this rearrangement resulted in some disorder within the genome: gene might be altered (mutation), lost (unfound), converted (e.g. location change from A^n^ to C^n^ in *B. napus*), chromosomal location might be dissimilar with parents (e.g. parental gene on A^r^2 and descendant gene on A^n^9) [19, 55]. Besides, duplication event also occurred, which is usually the primary explanation of new genes emergence [56, 57]. Duplicated genes resulted in function divergence and affected the evolution [58, 59]. Otherwise, hybridization followed by genome duplication in *Brassica* engendered new species like *B. napus* which is a tetraploid of *B. rapa* and *B. oleracea* [16, 60, 61]. This is called “polyploidization”. The higher amount of gene copies observed in *B. napus* was normal as the consequence of polyploidization. During this polyploidization, chromosome rearrangement occurred in *B. napus* resulting in loss of genes compared to *B. rapa* and *B. oleracea* [19]. It was affirmed that loss of genes on A^n^ genome might be replaced by homologous genes on C^n^ genome, and reversely [19], but this involved seven genes only in the present study. Also, it was intriguing that more gene insertions were found in C^n^ genome of *B. napus*. Besides, the reference genome “*Darmor-bzh”* which was used for the sequencing of *B. napus* was not derived from hybridization of the reference genomes used for the sequencing of *B. rapa (Chiifu-401)* [62] and *B. oleracea (Capitata)* [63], so this might explain also the blurred reason of genes distribution in *B. napus* of the present study. Additionally, *B. rapa* and *B. oleracea* are vegetables and *B. napus* is an oil crop, thus, more copy number of oil related genes were normally found in *B. napus*. Furthermore, as mentioned earlier, long years of evolution and artificial selection have resulted in difference between the parental A^r^ genome of *B. rapa*, C^o^ genome of *B. oleracea* and the descendant A^n^ and C^n^ genomes of *B. napus* [10]. Otherwise, in the present study, more gene copies were found in *B. napus,* and paralogous genes might be subfunctionalized or neofunctionalized, as these phenomena are commonly seen in newly emerged genes [64, 65].

### Environments influenced also the QTL alignment map which revealed fixed QTLs for particular environment

In the current study, a QTL alignment map was generated according to the related molecular markers and they were aligned on the physical map of *Darmor-bzh*, QTLs detected in diverse populations had dissimilar location on the physical map of *Darmor-bzh.* It is not new that genotype and environmental characteristics influence QTLs. Zhu and Zhao (2007) emphasized that factors influencing the number of detected QTLs in one given population depends on the genetic variation between the two parents, the type (DH or RIL) and size of the population, and the number of environments used [53]. The populations used in our study were derived from different parental lines, we both used DH and RIL, but the size of population was different and the number of environments as well (Table 2), thus, this explained the dissimilarity in QTLs detected in each population, which in consequence affected our QTLs alignment map. More genetic variation involved in the trait is one of the bases that more relative QTLs can be identified. Also, larger number of lines studied could allow higher detection of the genetic loci related to the trait variation [66]. In this study, FA-QTLs and OC-QTLs from nine populations of *B. napus,* with dissimilar environments and genotype background were used. First, looking at the parental lines of populations used in this study (Table 2), it is obvious that they have no direct relationship. In our study, we compared QTLs from these nine different populations, QTLs were for the same traits, but obtained in totally different genetic backgrounds (with no parent in common). It is a very good approach for detecting hot spot genomic regions associated with the traits, but not always accurate for validating exact QTL. These nine populations were produced from hybridization of different varieties of *B. napus*: *DY* is a hybrid of European and Korean [37], *SG* and *TN* are hybrids of European and Chinese [35, 67], *Z5, KN* and *M201XM202* are Chinese [42, 48, 68], *SO* is European [69] and *PT* is Canadian [70]. Genesis of variety within a species initiates in natural selection which enables species to settle into specific environmental pressures. Inherited variations occur in natural selection, individuals with suitable traits survive and reproduce better than the others, and the genetic information are inherited by their descendant. Pressures might come from biotic and/or abiotic factors [71, 72]. In selective breeding, artificial selection allows the production of new varieties according to the desired traits. Although varieties are phylogenetically divergent, overlapping QTLs could be detected, due also to the influence of environments.

**Table 2:** List of populations used to establish a map aligning QTLs for FA and OC traits

	Populations	Type	Environments	Lines	OC (%)	OC-QTLs	FA-QTLs	References
*Darmor-bzh x Yudal*	*DY*	SW	France	445 DH	38–54	14	–	[37]
*Rapid x NSL96/25*	*RNSL*	W	France, Germany	242 DH	35.7–50	10	–	[37]
*zy036 × 51,070*	*Z5*	S, W, SW	China	92 DH	30.85–51.30	12	–	[39]
*Sollux x Gaoyou*	*SG*	W, SW	Germany, China	282 DH	35.6–57	9	–	[40, 41]
*Tapidor x Ningyou7*	*TN*	S, W	China	404 DH	33.8–50.9	41	–	[44]
*Tapidor x Ningyou7*	*TN*	S, W, SW	China	202 DH	–	–	72	[45]
*KenC-8 x N53–2*	*KN*	S, W, SW	China	348 DH	42–47.6	24	–	[42]
				300 DH	35–57.5	67	204	[43]
*M201XM202*	*M201XM202*	W	China	149 RIL	29.16–48.93	15	–	[48]
*Sansibar x Oase*	*SO*	W	Germany, Sweden	226 DH	41.2–48.6	5	16	[46]
*Polo x Topas*	*PT*	W	Canada	156 DH	31.5–55.5	14	131	[47]

*Abbreviations*: *S* (Spring), *W* (Winter), *SW* (Semi-winter), *DH* (Double haploid), *RIL* (Recombinant inbred lines)

Phenotypic variation of trait could be influenced by genetic and environmental factors [53], but overlapping QTLs underlined conserved regions on the genome which were responsible for trait variation. In the present results, populations which were cultivated in similar environments had more overlapping QTLs, such as *KN* and *TN* populations. Overlapping QTLs in these populations might be fixed QTLs for Chinese environment. The Canadian line also had few QTLs overlapping with the others, these QTLs were rather specific. However, the poor overlapping QTLs found in European cultivated populations were intriguing. Also, we found three overlapping QTLs in A1 and A3 regions, involving populations of dissimilar environments, these might correspond to enrichment region of associated gene variations. It is also probable that the populations share common ancestors but this needs to be verified.

In the current study, we found specified QTLs for independent populations. They could not overlap with other QTLs due probably to various factors, such as power of detection, density of genetic map, difference between parents, environments, type and size of population (Table 2). Thus, concerning these available populations, comprehensive assessment of the genetic background, or selection history of the parental lines that led to the roles of those specific QTLs, or in specific environment have not been made yet. Currently, more high density maps are being constructed and published, based on the same population. We expect in the future that the results obtained in our study would serve to assess the genetic background and evolution of rapeseed.

Otherwise, we compared our results with previously published consensus map and due to the difference of markers used, comparable results could be obtained only with those published by Wang et al. (2013). In fact, they built a consensus map based on common markers, for oil content QTL including one RIL and seven DH populations *GS/05, GS/12, DY, RNSL, Z5, TN* and *KN*. Six overlapping QTLs were detected: one on A1 chromosome (*KN-qOC-A1–1* overlapping with *TN-qOC-A1* and *DY-qOC-A1–2*); one on A2 (*KN-qOC-A2–3* overlapping with *DY- qOC-A2–2*), one on A8 (*KN-qOC-A8–1* overlapping with *TN-qOC-A8* and *RNSL-qOC-A8*); one on C3 (*KN-qOC-C3–2* overlapping with *RNSL-qOC-C3*); and two on C9 (*KN-qOC-C9–2* and *Z5-qOC-C9–1, KN-qOC-C9–3* and *Z5-qOC-C9–2*) [42]. By comparing markers interval, BRAS068 on chromosome C3 was aside of overlapping *KN-qOC-C3–2* and *RNSL-qOC-C3* of Wang et al. (2013) map and *qOC-C3–1-TN* and *qOC-C3–2-KN* of the present results. A fragile comparison with the consensus map made by Wang et al. (2013) resulted in poor findings, due to the limitation imposed by the difference of markers. Other approaches such as using common markers might lead to more discovery.

In the present study, one locus on the C3 chromosome (53.75 Mb to 58.29 Mb) might affect six traits (C16:0, C18:0, C18:1, C18:2, C20:0, and C22:1). Any changes within this locus, harsh or beneficial, could affect these traits, it is interesting to tailor more than one desirable trait at the same time. Wang et al. (2014) investigated on genetic changes on current breeding of *B. napus*, and discovered that C genome (57.15 Mb) had extended breeding regions compared to A genome (16.80 Mb), but also C genome might have contributed to more valued alleles to produce elite traits [73]. This might explain the fact that more gene insertions were found on C genome in this study, but also the region on C3 which might affect multiple traits in our study. This region on C3 is then a favorable region to develop for rapeseed breeding. Further analysis could help into understanding of varietal characteristic of rapeseed, which is useful for the selection of population for breeding, for instance, use of the current results to compare with other populations not used in this study.

### New potential candidate genes were found, which might affect multiple traits

Candidate gene investigation allows the identification of valuable genes associated to agriculturally and economically quantitative traits. Precise genetic architecture, distribution and interaction of loci that affect variation, permit the understanding of their effect on phenotypic variation [53]. In the present study, 162 QTLs underlined 218 candidate genes of *B. napus* which belonged to 45 families, they were homologous of 91 *A. thaliana* genes; and 76 among these candidate genes were found in 57 overlapping QTLs intervals. They were located in the QTL confidence intervals for C16:0, C18:0, C18:1, C18:2, C18:3, C20:0, C22:1, UFA, OC traits. In general, identification of candidate genes can be done via genome wide association studies (GWAS), linkage studies, expression studies but also it needs a prior understanding of the biological pathway. Lou et al. (2006) affirmed that combining the candidate genes with linkage studies could effectively enhance the accuracy [74].

In this study, we preselected the candidate genes by their correlation with the studied traits. The prior knowledge of the biological function of future candidate genes is important because many genes could be identified, especially if the study relies on position-dependent strategy, but the better are those which have functional consequences on the biological pathway or have close connection to the studied traits [53]. Thus, we both used function and position-dependent strategy to identify the potential candidate genes. The identified candidate genes were just putative causal genes, only experimental approaches can validate the accuracy of these results. LT gene BnaA08g12720D was a candidate gene on A3, which might affect C18:1 and oil. The positive correlation between C18:1 and oil content has been demonstrated in multiple studies [29, 75, 76]. Oleic acid (C18:1) is an omega-9 fatty acid which composes naturally the animal and vegetable oils. Sales-Campos et al. (2012) reviewed the effect of this monounsaturated fatty acid on health, including its beneficial usage on wounds and inflammation, on regulation of blood pressure, on immunity system and on cancer healing process [77]. To take advantage of these, LT would be an ideal choice for altering C18:1 and oil at the same time, if it is proven experimentally to affect these traits. In *SO* map, Teh and Möllers (2016) identified FAD2 overlapping with QTLs for C18:1 and C18:3 on chromosome A1 by alignment with *B. rapa* [46]; however, the present study did not underline candidate genes in this QTL region. By comparing the present results with those published by Wang et al. (2015) (*TN* population only), divergence in findings was obvious. In fact, they identified 234 genes homologues of *A. thaliana* in 47 QTLs, involved in fatty acid metabolism [45]. In our study, 32 *A. thaliana* genes were found to be similar to those detected by Wang et al. (2015). Remaining 59 homologous genes were new candidate genes. Among these 32 similar candidate genes, 18 genes were potential genes for the same traits discovered in both our analysis and Wang et al. (2015) analysis (Additional file 5: Table S5). However, divergence in some *B. napus* genes were perceived, for instance, FATB-At1g08510 was also seen in our analysis, but homologue gene in *B. napus* (BnaA08g26890D) failed to be a candidate gene. Wang et al. (2015) also found FAD2, FAD3, FAE, LEC, FUS3, WRI1 and ABI genes as potential genes. Additionally, the genes ACC2, FAE1 and LPAAT were found in all FA-QTL intervals, while LEC1, LEC2, ACC2, and KASIII underlined C16:0. However, in our results, ACC2 were underlined by C18:2-QTL and C18:3-QTL only, FAE were involved in all traits unless C18:3 (but this was similar to the result of Wang et al. (2015), in which FAE fell into the CI of qC3–2 involving all traits unless C18:3). Besides, LPAAT were found in QTL interval of C16:0, C18:1, C18:2, C18:3 and OC, whereas LEC were inside QTL for C16:0, C18:3 and OC; and KASIII were found in the QTLs of C18:3 and OC traits. Otherwise, several of the candidate genes identified in our study have already demonstrated their effectiveness in enhancing oil content. As mentioned earlier, the abilities of DGAT, GPDH and GPAT to improve oil content have been demonstrated [32, 33], DGAT-BnaA08g03400D and GPAT-BnaA08g06960D both fell inside QTL for oil content in our analysis. In the present time, homologues in *B. napus* must be undergoing multiple analyses for similar or new functions discovery.

### Candidate genes could be affected at multilevel of oil formation

Focusing on network interaction and pathway analyses, genes belonging to KASI, KASII, LPAAT, GPAT, PDAT, ACC, ACP interacted most with other genes. They were found in multiple populations unless PDAT and ACC and could affect oil traits unless ACC. Similar analysis were made by Wang et al. (2015) in which direct or indirect effect of transcription factors over the genes were highlighted. In the present results, poor connection between transcription factors and candidate genes were found. As mentioned earlier, seed oil content is a trait controlled by a versatile genetic structure and also influenced by the environment [26, 27]. Association of these detected candidate genes, which obviously might depend on the ten above-mentioned key genes, underline structure that run the overall system. It has been affirmed that structure and dynamism of genetic regulatory networks influence quantitative traits [78] and genes are responsible for QTLs, affecting genetic variation of traits [79]. Because our analysis took in consideration nine populations at the same time, separated analysis of individual population might lead to different results. Also, since QTLs were dissimilar in the nine studied populations, it is possible that the genes involved in the system were different, which affected QTLs and traits in consequences. Previously, we found that some genes could affect one given trait in a population, and affect another trait in another population, this is the case of ACBP gene BnaA03g29000D which was found in QTL interval of C16:0 in *PT* population, but in oil content in *DY, TN* and *Z5* populations. Finally, since candidate genes interacted strongly with ten key genes of FA and TAG biosyntheses, traits were then affected at multilevel of oil formation.

### Advantages and limitations of our study

The current study aimed to identify overlapping QTLs from diverse populations of different environments background, and related potential candidate genes that might affect fatty acid profile and oil content in *B. napus*. We used function and position-based strategy to identify the candidate genes. This strategy allowed to detect QTL hotspots which maybe enrichment regions of gene variation involved in fatty acids and oil biosyntheses. This strategy also offered the advantage of eliminating genes inside QTL region which were not involved in fatty acid and oil biosyntheses. Besides, building the QTL alignment map allowed to make possible and easier the comparison of QTLs identified in diverse populations, which could be combined in one map, despite the difference of markers. Also, related candidate genes could be discovered. It was regrettable that some QTLs could not be settled on the map due to missing marker sequences. Additionally, the map helped us to verify stable QTLs, which could help us to focus on valuable loci for fine mapping. Although stable QTLs were not confirmed yet, we could discover QTL enrichment regions which also gave us clues in genetic mechanism of close linkage of each trait or trait with trait, and discover easy variant area and conservative area. Finally, we analyzed the interaction network of candidate genes in order to understand their interaction, their influence on each other. We also built a metabolism pathway highlighting the discovered candidate genes, and the traits that they might affect. STRING and Cytoscape_V3.2.1 offered a simple and easy way to analyze and visualize gene interaction, they are commonly used for such analysis actually, but the fact that *B. napus* and *B. oleracea* is still missing on STRING Database, so that orthologous genes in *A. thaliana* were used for the analysis, it is probable that results obtained were not accurate.

## Conclusion

In conclusion, the present study allowed to build a QTL alignment map with diverse populations which could serve as reference to study the genome profile of *B. napus*. New potential genes emerged which need experimental approach for authentication. We offered new useful tracks for the selection of population or/and selection of interesting genes for breeding improvement purpose. As perspectives, we suggest the development of functional markers based on our results. Also, since the candidate genes were detected by using of the reference genome “*Darmor-bzh”,* it would be better to test the accuracy of our results in other population.

## Methods

### Identification of *Brassica* genes involved in FA synthesis and TAG formation and gene synteny analysis

FA are synthesized in the plastid and TAG are formed in the ER. In the present study, we took in consideration the genes related to these biosyntheses for an afterward detection of potential candidate genes for oil improvement. Thus, *A. thaliana* genes related to these biosyntheses were acquired from the website ARALIP (http://aralip.plantbiology.msu.edu/) and TAIR (www.arabidopsis.org) [80]. *Brassica* genes were identified based on homology to *A. thaliana* genes, with a score > 80, by using blastn (using *A. thaliana* nucleotides sequence) on *Brassica* Database (http://brassicadb.org/) [81] to get *B. rapa* and *B. oleracea* homologous genes; and browser (using *B. rapa* and *B. oleracea* gene names) on *Brassica napus* genome resource (http://www.genoscope.cns.fr/brassicanapus/) [19] to get *B. napus* homologous genes. The genes synteny was built with Circos software [82]: *B. napus* genes were linked to their homologous genes in *B. rapa* and *B. oleracea.*

### Identification of overlapping QTLs for FA and OC traits, and detection of potential candidate genes

OC-QTLs and FA-QTLs from nine previously reported populations were aligned into one map for comparison: *DY (‘Darmor-bzh’ × ‘Yudal’)* [37], *RNSL (‘Rapid’ × ‘NSL96/25’)* [37], *Z5 (‘zy036’ × ‘51,070’)* [39], *SG (‘Sollux’ × ‘Gaoyou’)* [40, 41], *KN (‘KenC-8’ × ‘N53–2’)* [42, 43], *TN (‘Tapidor’ × ‘Ningyou7’)* [44, 45], *SO (Sansibar × Oase)* [46], *PT (Polo × Topas)* [47] and *M201 × M202* [48]. The QTLs were projected onto the physical map of the reference genome “*Darmor-bzh”* and the position of related flanking markers were identified by using of E-PCR [83, 84]. First, the markers intervals were taken from an area less than 3 cM from the linkage map. Second, those markers’ primer sequences were acquired from related published papers. Then, by using E-PCR [83, 84], their positions on the physical map of *B. napus “Darmor-bzh”* could be deduced. Finally, these markers could be aligned on the physical map and the region inside two positioned markers was the QTL region. QTLs of which marker sequences were missing, or could not be placed on the corresponding chromosome were removed from this analysis. The studied traits were C16:0, C18:0, C18:1, C18:2, C18:3, C20:0, C20:1, C22:0, C22:1, combined unsaturated FA (UFA) and oil content (OC). The map was built with Circos software [82]. QTLs were renamed as “*q-trait-chromosome-population*” for uniformity, if many QTLs were detected on the same chromosome, the number of order was added to the name, e.g.: *qOC-A1–2-TN* referred to the second OC-QTL from *TN* population on chromosome A1. Overlapping QTLs were QTLs from two or more populations that were located in the same region, and potential candidate genes were genes located inside a QTL region.

### Gene interaction network and metabolism pathway analyses

In order to study the interaction between candidate genes, STRING was used (http://string-db.org/). STRING is a well-known database widely used to predict interactions (physical and functional) in proteins [85] which was then suitable for our study. *B. napus* and *B. oleracea* genes cannot be directly used for network analysis in STRING Database due to unavailability. Thus, interaction of candidate genes was studied by using of orthologous genes in *A. thaliana*. The orthologous genes of *A. thaliana* were submitted to STRING search using protein names and *A. thaliana* as organism. Then the resulting network was visualized with Cytoscape_V3.2.1 [86]. The interaction was classified according to the degree layout (DL). More edges indicate more interaction with other genes. Then, potential metabolism pathway was manually constructed referring to the Acyl-Lipid Metabolism of The *Arabidopsis* Book and the website ARALIP (http://aralip.plantbiology.msu.edu/), gene families were placed according to their roles in oil formation [21], and corresponding gene names were summarized on Additional file 4: Table S4.

## Additional file

Additional file 1: Table S1. Genes involved in FA biosynthesis and TAG formation in *A. thaliana, B. rapa, B. oleracea* and *B. napus*. (XLSX 39 kb)
Additional file 2: Table S2. QTLs for FA and OC in nine populations, mapped to *B. napus “Darmor-bzh”* reference genome v4.1. (XLSX 33 kb)
Additional file 3: Table S3. Overlapping QTLs for FA and OC in nine populations, mapped to *B. napus “Darmor-bzh”* reference genome v4.1. (XLSX 21 kb)
Additional file 4: Table S4. Candidate genes detected in QTL intervals from nine populations, with their respective homolog in *A. thaliana*. (XLSX 31 kb)
Additional file 5: Table S5. Comparison with Wang et al. (2015) study, showing same *A. thaliana* orthologous genes of detected candidate genes *in B. napus*. (XLSX 10 kb)

## Abbreviations

AAPPT: Diacylglycerol Cholinephosphotransferase
ABCAT: ABC Acyl Transporter
ABI3: Homologous to the maize transcription factor Viviparous-1
ABI4: Abscisic Acid Insensitive (ABI) transcription factors
ACBP: Acyl-coA binding proteins
ACC: Acetyl-coA Carboxylase
ACP: Acyl Carrier Protein
ASIL1: Trihelix DNA Binding Family
CCT: Choline-Phosphate Cytidylyltransferase
CK: Choline Kinase
DGAT: Acyl-CoA: Diacylglycerol Acyltransferase
DHLAT: Dihydrolipoamide Acetyltransferase
ER: Enoyl-ACP Reductase
FAD2: Oleate Desaturase
FAD3: Linoleate Desaturase
FAE: Fatty acid elongase
FATA: Acyl-ACP Thioesterase A
FATB: Acyl-ACP Thioesterase B
FUS3: Transcriptional factor
GPAT: Glycerol-3-Phosphate Acyltransferase
GPDH: NAD-dependent Glycerol-3-Phosphate Dehydrogenase
HAD: Hydroxyacyl-ACP Dehydrase
HAS: Holo-ACP Synthase
HSI2/VAL1: a member of a novel family of B3 domain proteins
HSL1/VAL2: a member of a novel family of B3 domain proteins
KAR: Ketoacyl-ACP Reductase
KASI: Ketoacyl-ACP Synthase I
KASII: Ketoacyl-ACP Synthase II
KASIII: Ketoacyl-ACP Synthase III
LCAS: Long-Chain Acyl-CoA Synthetase
LEC: Transcription factor/activator
LPAAT: 1-Acylglycerol-3-Phosphate Acyltransferase
LPCAT: 1-Acylglycerol-3-Phosphocholine Acyltransferase, Lysophospholipid acyltransferase
LPD: Dihydrolipoamide Dehydrogenase
LS: Lipoate Synthase
LT: Lipoyltransferase
MCMT: Malonyl-CoA: ACP Malonyltransferase
PDAT: Phospholipid: Diacylglycerol Acyltransferase
PDCT: Phosphatidylcholine:diacylglycerol cholinephosphotransferase
PKL: a SWI / SWF nuclear-localized chromatin remodeling factor of the CHD3 group
PLA2: Phospholipase A2
PP: Phosphatidate Phosphatase
SAD: Stearoyl-ACP Desaturase
WRI1: WRINKLED1
α-CT: Alpha-carboxyltransferase
α-PDH: Alpha-Pyruvate Dehydrogenase
β-CT: Alpha-carboxyltransferase
β-PDH: Beta-Pyruvate Dehydrogenase
